# Identifying Polycentric Urban Structure Using the Minimum Cycle Basis of Road Network as Building Blocks

**DOI:** 10.3390/e27060618

**Published:** 2025-06-11

**Authors:** Yuanbiao Li, Tingyu Wang, Yu Zhao, Bo Yang

**Affiliations:** 1 Data Science Research Center, Kunming University of Science and Technology, Kunming 650500, China; liyb6755@163.com (Y.L.); tywang@stu.kust.edu.cn (T.W.); zhao774629876@163.com (Y.Z.); 2 Faculty of Science, Kunming University of Science and Technology, Kunming 650500, China

**Keywords:** urban road network, motif, the minimum cycle, power-law distribution

## Abstract

A graph’s minimum cycle basis is defined as the smallest collection of cycles that exhibit linear independence in the cycle space, serving as fundamental building blocks for constructing any cyclic structure within the graph. These bases are useful in various contexts, including the intricate analysis of electrical networks, structural engineering endeavors, chemical processes, and surface reconstruction techniques, etc. This study investigates the urban road networks of six Chinese cities to analyze their topological features, node centrality, and robustness (resilience to traffic disruptions) using motif analysis and minimum cycle bases methodologies. Some interesting conclusions are obtained: the frequency of motifs containing cycles exceeds that of random networks with equivalent degree sequences; the frequency distribution of minimum cycle lengths and surface areas obeys the power-law distribution. The cycle contribution rate is introduced to investigate the centrality of nodes within road networks, and has a significant impact on the total number of cycles in the robustness analysis. Finally, we construct two types of cycle-based dual networks for urban road networks by representing cycles as nodes and establishing edges between two cycles sharing a common node and edge, respectively. The results show that cycle-based dual networks exhibit small-world and scale-free properties. The research facilitates a comprehensive understanding of the cycle structure characteristics in urban road networks, thereby providing a theoretical foundation for both subsequent modeling endeavors of transportation networks and optimization strategies for existing road infrastructure.

## 1. Introduction

In recent years, researchers have embarked on a comprehensive exploration of various transportation networks, including railways [1,2], aviation [3,4], urban roads [5,6,7,8,9,10,11,12,13,14,15,16,17], and others [18,19,20]. As a vital component of a city, urban roads play an important role in the normal operation of urban functions [21,22]. However, with the acceleration of urbanization and the rapid surge in transportation demand, the complexity of urban road systems has increased significantly [23,24], and complex network theory has gained widespread acceptance in observing and analyzing this intricate structure [25,26,27].

In the study of urban road networks, researchers have explored the structural characteristics of urban roads from various perspectives, including self-similarity [5], connectivity [6], spatial layout [7], growth and evolution [8], topological structure [9,10,11,12,13], and robustness [14,15,16,17].

In terms of the node centrality and topological structure of urban road networks, researchers have conducted the following studies. Zhang et al. [9,10] studied the relationship between morphological features and topological characteristics, such as network degree centrality, network betweenness centrality, and network closeness centrality. Their findings revealed that betweenness centrality can effectively distinguish and describe different morphological road networks. Tsiotas et al. [11] analyzed the relationship between urban roads and space, discovering that the degree distribution and connectivity of road networks are influenced by urban spatial constraints. Jiang et al. [12] studied the degree characteristics of urban road networks and found a lack of degree correlation within the road networks. Shang et al. [13] examined the topological structure of urban road networks, including clustering coefficient and average path length, and found that the small-world characteristics of urban road networks are not significant.

Regarding the resilience of urban transportation systems, the following key findings have been validated. Firstly, the adoption of different attack strategies has a significant impact on network robustness. Liu et al. [14] studied the model of cascading failures in urban road networks, revealing that road intersections have the greatest impact on robustness. Furthermore, random attacks have a weaker impact on network robustness compared to targeted attacks. Secondly, there are differences in network robustness across different granularities. Duan et al. [15] modeled urban road networks based on three granularities: segment-based, stroke-based, and community-based. Their results showed that due to the similarity in degree distribution and topological structure among urban road networks, robustness tends to be consistent. However, structural differences across granularities lead to variations in robustness. Thirdly, the impact of attacks targeting different topological metrics on network robustness varies. Zhao et al. [16] employed a duality method [17] (which abstracts road segments as nodes in the road network and converts connectivity relationships, such as intersections, into edges in the network) to transform urban road networks into topological graphs for robustness analysis. Their findings indicated that node degree has the greatest impact on robustness, followed by node betweenness and edge betweenness.

Milo et al. [28,29] proposed the concept of network motifs, which are small subgraphs of a network formed by a limited number of nodes arranged in a specific topological structure. In the context of road networks, motifs refer to road structural units with specific spatial configurations and traffic characteristics. These motifs appear more frequently in urban road networks than in random networks with the same degree sequence. The design and layout of these motifs directly impact the traffic flow efficiency of the road network. An optimal layout of motifs can make the road network more compact and connected, enhancing the accessibility and convenience of the city [30].

Urban road network analysis using motif methods remains underexplored, particularly regarding Z-scores (which quantify the statistical significance of recurring substructures by comparing their observed frequency to random network expectations) with comparative analyses across motif types still lacking [30,31,32]. To address this gap, we conducted a detailed analysis of urban road networks using motif methods and obtained the Z-scores for various motifs. The results revealed that motifs with cycles exhibited positive Z-scores, indicating that their frequency of occurrence was significantly higher than in random networks with the same degree sequence. In other words, cycles are ubiquitous in urban road networks, and thus they can serve as fundamental building blocks for studying urban road systems.

In the field of network analysis, the significance of cycles has garnered attention, and some exploratory research has been conducted (e.g., the distribution of cycles of different sizes in real networks and artificial networks [33,34,35,36,37,38,39,40]). Zhang et al. [41] proposed the cycle nodes ratio (the ratio of the number of nodes belonging to cycles to the number of total nodes) for network classification. Fan et al. [42] proposed the use of the cycle ratio (the summation of co-cycle rates among nodes sharing the same cycle with the target node) as a metric to assess the importance of nodes, comparing it with the results obtained from degree, H-index, and coreness. Experiments on real-world networks suggest that the cycle ratio contains rich information in addition to well-known benchmark indices. However, we measured the importance of nodes based on their cycle ratio in the road network. The results showed that nodes having higher cycle degrees (defined as the number of minimum cycles related to the node) within large cycles were prone to having a higher cycle ratio; however, this method could not effectively identify the central nodes in the road network. To solve this problem, we will introduce the concept of cycle contribution rate (taking into account both the quantity and length of cycles associated with each node) to assess node importance. The reason lies in the calculation differences between the cycle ratio and the cycle contribution rate. The former sums over all co-cyclic nodes in the entire network, amplifying the influence of large cycles; while the latter only accumulates contributions from directly associated cycles, emphasizing that small cycles contribute more significantly than large ones. In summary, existing network analysis methods based on cycles exhibit a lack of systematization and mathematical rigor. Notably, in graph theory, the concept of a minimum cycle basis has been proposed and applied to the analysis of small-scale networks [43,44,45,46]. For any graph, all cycles within it collectively form a cycle space. By identifying a set of linearly independent cycles within this cycle space, a minimal cycle basis can be constructed. All cycles in the network can then be expressed through this minimum cycle basis, much like how all vectors in a vector space can be represented by a set of basis vectors.

Building upon that foundation, we integrate existing cycle-related research with the theory of minimum cycle bases to develop a comprehensive methodology for road network analysis. Specifically, first, we conduct motif analysis on road networks to verify the widespread existence of cycle structures in road networks. Then, based on the minimum cycle basis, we investigate the topological characteristics (including the total number of cycles in the minimum cycle basis, the edge length distribution, and the surface area distribution of the basis, among other metrics), node centrality (including degree, cycle degree, cycle ratio and cycle contribution rate), and robustness (the relative size of the maximum connected subgraph, average cycle length in the minimal cycle basis, and total cycle count in the minimal cycle basis are measured as functions of node removal ratio under different removal strategies (degree, betweenness, cycle degree, cycle ratio, and cycle contribution rate) during progressive node removal processes) of urban road networks, respectively. Finally, we constructed two types of cycle-based dual networks by treating the minimum cycle basis as nodes and establishing connections based on the shared one node and one edge between cycles, respectively. The topological properties of the cycle-based dual network are analyzed, including degree distribution, clustering coefficient, average path length and network diameter.

The structure of this paper is arranged as follows: In Section 2, we provide a detailed introduction to the minimum cycle basis model, robustness analysis methods, and the cycle-based dual network model. In Section 3, we present a comprehensive analysis and further discussion of the research findings in urban road networks. Finally, in Section 4, we summarize and draw some conclusions.

## 2. Model and Method

### 2.1. Motif and Z-Score

**Figure 1:**
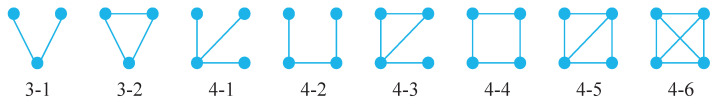
The schematic diagram of motifs consisting of three or four nodes in the undirected network.

Motif is a fundamental pattern of repeated interactions in networks that occur significantly more frequently than in random networks with the same number of nodes and edges. Eight motifs of the undirected network are shown in Figure 1, including two three-node motifs and six four-node motifs.

To measure the importance of motifs in real-world networks, it is necessary to generate a large number of random networks that are consistent with the degree distribution of real-world networks. To compare the frequency of motifs in real-world networks with those in random networks, the Z-score is introduced. For a motif *i*, the Z-score is calculated as [47]:

(1) $$\begin{array}{c} Z_{i}=\frac{N_{real_{i}}-\langle N_{rand_{i}}\rangle }{\sigma_{rand_{i}}}, \end{array}$$

where $N_{real_{i}}$ is the frequency of occurrence of motif *i* in the real-world network $\langle N_{rand_{i}}\rangle$ and $\sigma_{rand_{i}}$ is obtained from 1000 different random networks with the same degree distribution as the real-world network, representing the average frequency and standard deviation, respectively. When $N_{real_{i}}>\langle N_{rand_{i}}\rangle$, the Z-score is positive, which indicates that the motif occurs more frequently in the real-world network. When $N_{real_{i}}<\langle N_{rand_{i}}\rangle$, the Z-score is negative, which indicates that the motif occurs less frequently in the real-world network.

### 2.2. Observables Based on Cycle

For a given undirected network G=(V,E), $V=\{v_{1},v_{2},\;\ldots \;,v_{N}\}$ represents the set of *N* nodes, and *E* represents the set of edges, which are unordered pairs of elements of *V*. A cycle $O_{i}:v_{1}v_{2}\ldots v_{k-1}v_{k}v_{1}$ is a subgraph where every vertex has an even degree, and its length is the number of its edges. Cycles in a graph generate the cycle space of *G*. A cycle basis of *G* is defined as a maximal set of linearly independent cycles. The dimension of the cycle space is M−N+κ(G), where κ(G) is the number of connected components of *G*. For an unweighted graph, the cycle basis where the sum of the length of the cycles is minimum is called a minimum cycle basis of *G* [43,44,45,46]. Specifically, the minimum cycle of a planar graph is the set of bounded faces [45,46].

For a node $v_{i}$, the degree is $k_{i}$, and the set of cycles associated with $v_{i}$ is presented as $C_{i}=\left\{C_{i1},C_{i2},\;\ldots ,\;C_{in_{i}}\right\}$, where $n_{i}$, called cycle degree, is the size of $C_{i}$. That is to say, the cycle degree of node $v_{i}$ refers to the number of minimum cycles associated with node $v_{i}$. The length of each cycle in $C_{i}$ is denoted by $l_{i1}^{c},l_{i2}^{c},$$\ldots ,\;l_{in_{i}}^{c}$. In order to measure the importance of nodes in the urban road network, we propose an observation based on the cycle, named cycle contribution rate, which is defined as follows:

(2) $$\begin{array}{c} \rho_{i}=\sum_{j=1}^{n_{i}}\frac{1}{l_{ij}^{c}}. \end{array}$$

The cycle contribution rate reflects the idea that a node’s significance is determined by both the number and the length of cycles it participates in. A high rate signifies dense involvement in numerous short cycles, suggesting a central network role, while a low rate implies sparse participation in longer cycles, indicating a more peripheral position. This dual metric thus captures structural centrality through cyclical connectivity patterns.

In Ref. [36], the cycle ratio is proposed as an effective measurement based on the minimum cycles to examine the importance of nodes. Similarly, we propose that the cycle ratio $r_{i}$ based on the minimum cycle basis is defined as follows:

(3) $$\begin{array}{c} r_{i}=\sum_{j}\frac{n_{i}^{j}}{n_{j}}, \end{array}$$

where $n_{i}^{j}$ represents the total number of cycles associated with nodes $v_{i}$ and $v_{j}$ simultaneously; the cycle degree $n_{j}$ refers to the number of minimum cycles associated with node $v_{j}$. A high cycle ratio suggests that the node is embedded within a large cycle and exhibits extensive cyclic associations, whereas a low cycle ratio implies its presence in a small cycle with limited cyclic connectivity.

**Figure 2:**
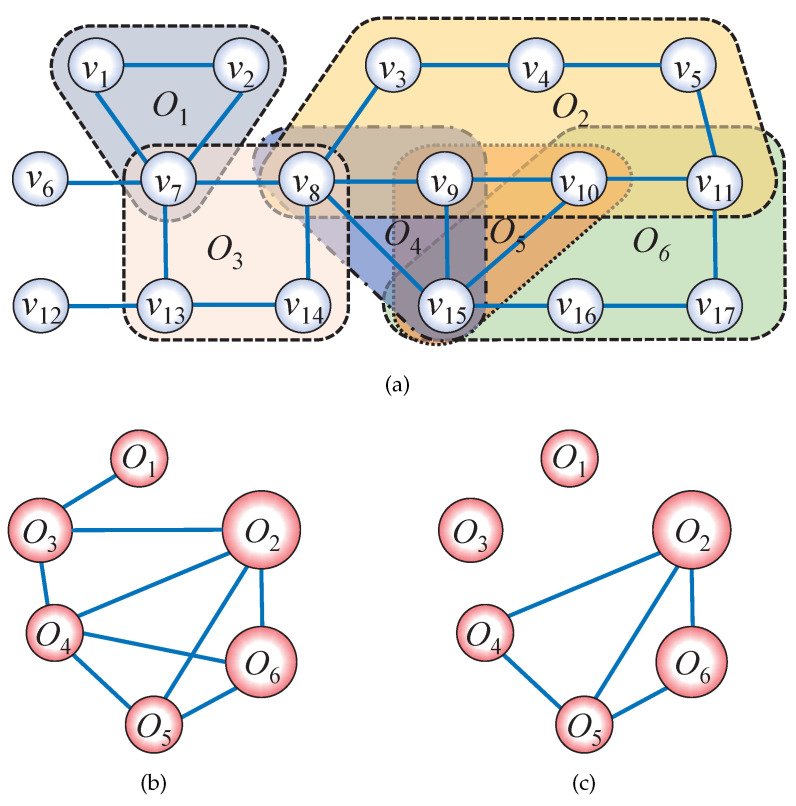
An example network and its cycle-based dual networks. (**a**) There are six minimum cycles: $O_{1}:v_{1}v_{2}v_{7}v_{1}$, $O_{2}:v_{3}v_{4}v_{5}v_{11}v_{10}v_{9}v_{8}v_{3}$, $O_{3}:v_{7}v_{8}v_{14}v_{13}v_{7}$, $O_{4}:v_{8}v_{9}v_{15}v_{8}$, $O_{5}:v_{9}v_{10}v_{15}v_{9}$ and $O_{6}:v_{10}v_{11}v_{17}v_{16}v_{15}v_{10}$. (**b**) The cycle-based dual network that establishes connections by sharing one node between the minimum cycle basis. (**c**) The cycle-based dual network that establishes connections by sharing one edge between the minimum cycle basis.

Figure 2 is a schematic diagram of the basic form and cycle characteristics of a network. Table 1 presents the degree, betweenness centrality, cycle degree, cycle ratio and cycle contribution rate of a node shown in Figure 2a.

For an undirected network *G*, define the set of cycles as $C^{G}={⋃}_{i=1}^{N}C_{i}=$$\left\{C_{1}^{G},C_{1}^{G},\;\ldots \;,C_{N^{o}}^{G}\right\}$, where $N^{o}$ represents the total number of the minimum cycle basis in the network *G*, and the corresponding minimum cycle basis length is $l_{1}^{G},l_{2}^{G},\;\ldots \;,l_{N^{o}}^{G}$, then the average length of the minimum cycle basis $\langle L^{c}\rangle$ is defined as follows:

(4) $$\begin{array}{c} \langle L^{c}\rangle =\frac{1}{N^{o}}\sum_{i=1}^{N^{o}}l_{i}^{G}. \end{array}$$

The average length of a minimum cycle basis reflects the mean value of all cycle lengths within it. A higher value indicates a larger proportion of longer cycles, while a lower value suggests a greater quantity of shorter cycles.

To investigate the overall properties of the network, we conducted a robustness analysis, which involves sequentially removing nodes from the network according to certain rules. Six strategies are employed to remove nodes from the network based on randomness, degree, betweenness, cycle degree, cycle ratio, and cycle contribution rate. The relative size of the maximum connected subgraph *S*, the average length $\langle L^{c}\rangle$ and the total number $N^{o}$ of minimum cycles in the network are selected as evaluation metrics to analyze the network robustness. *S* is denoted as follows:

(5) $$\begin{array}{c} S=\frac{N^{\prime }}{N}, \end{array}$$

where *N* represents the total number of nodes in the initial network, and $N^{\prime }$ represents the total number of nodes in the maximum connected subgraph after node removal.

### 2.3. Cycle-Based Dual Network

To analyze the interrelation among individual cycles, a cycle-based dual network is constructed in this paper, where each cycle is treated as a network node, and if two cycles share common nodes, a corresponding edge is established. Two cycle-based dual networks in Figure 2a are constructed as Figure 2b and Figure 2c where each pair of cycles shares one node and one edge, respectively.

## 3. Results and Discussion

### 3.1. Data Introduction and Preprocessing

**Figure 3:**
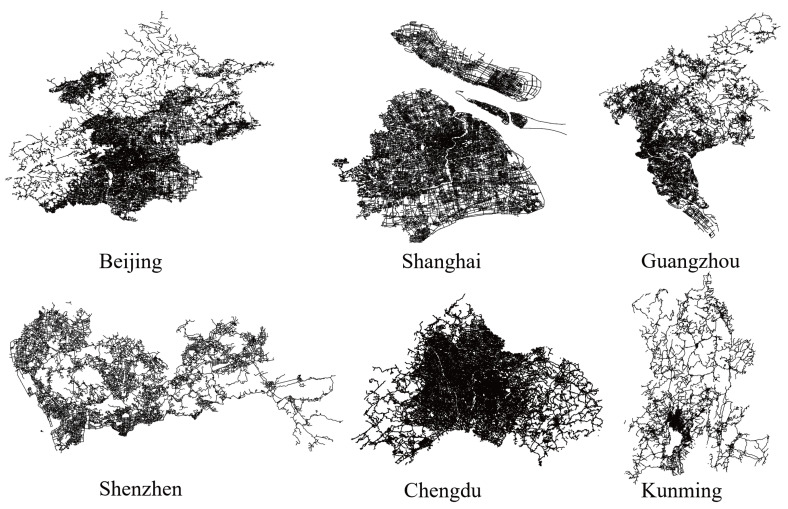
Experimental urban road networks.

Population, economy and comprehensive strength are the key indicators to evaluate urban development. This study focuses on six cities in China (Beijing, Shanghai, Guangzhou, Shenzhen, Chengdu and Kunming) to explore the potential information of urban road networks. Figure 3 visualizes the geographical morphology of the six road networks.

The urban road network data used in this study are obtained from the official OpenStreetMap (OSM) website (www.openstreetmap.org). The OSM database stores a huge environmental spatial dataset with very rich metadata covering the major cities of the world. The OSM network data of six cities in China were obtained on 20 September 2023 in the shp vector data format and the projection coordinate system was the WGS_1984_Mercator coordinate system.

OSM road data have good integrity, but the initial data are rough. In order to improve the quality of road network data and the accuracy of experimental results, data preprocessing is essential. In this paper, data preprocessing using ArcGIS 10.0 includes verifying data consistency, eliminating overlapping paths and invalid data, converting double lines into single lines, and finally converting single lines into a simple graph structure. In addition, the topology of the network needs to be checked, and isolated roads must be removed to ensure network connectivity.

We choose four road categories to model from OSM road data, which include main roads, secondary trunk roads, branch roads and internal roads. The main road is the backbone of the urban road network, which includes primary and secondary field in OSM. The secondary trunk road is a regional traffic trunk road in the city, which includes a tertiary field in OSM. The branch road is the connection line of the secondary trunk road connecting each residential area, which includes residential and unclassified fields in OSM. The internal road includes bridleway, living_street, path, and service field in OSM.

**Figure 4:**
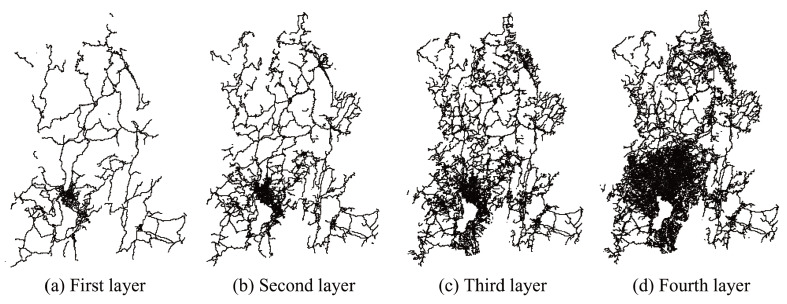
Different layers of urban road networks in Kunming.

In the modeling process, we divide the road network into four levels, namely the first layer network, the second layer network, the third layer network, and the fourth layer network. The first layer network only considers main roads, the second layer network adds secondary trunk roads on the basis of the first layer network, the third layer network introduces branch roads on the basis of the second layer network, and the fourth layer network adds internal roads on the basis of the third layer network. Figure 4 shows the spatial distribution of the four-layer road network.

**Figure 5:**
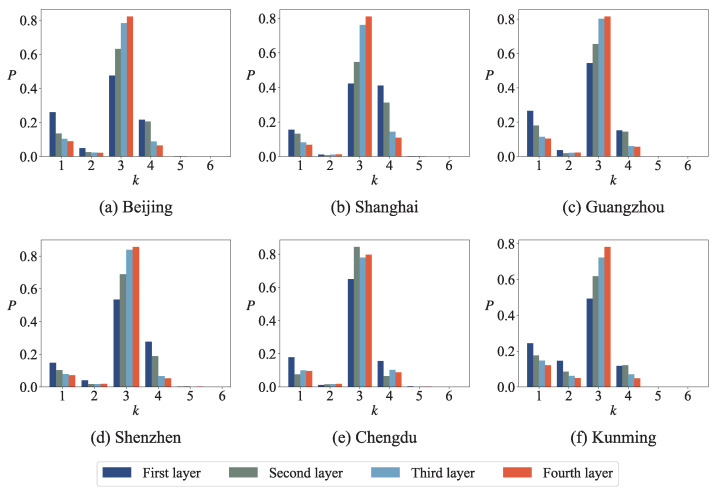
The degree distribution of urban road network at different layers. In all plots, the *x*-axis label *k* represents the degree of nodes and the *y*-axis label *P* represents the proportion of each part.

Figure 5 shows the degree distribution of the urban road network at different layers. It can be seen that the degree distribution of the urban road network is discrete; only a few values can be obtained. In each layer of the network, nodes with degrees of 3 occupy a larger proportion, followed by nodes with degrees of 4 and 1; that is, T-shaped roads account for a larger proportion in the network, and there are also some intersections and damaged roads. It is well-known that degree distribution plays a significant role in various network fields, such as vital nodes identification, community detection, and link prediction. However, the degree distribution of roadway networks contains relatively limited information, which is unfavorable for the analysis and modeling of issues related to roadway networks. Therefore, in the following, we will attempt to analyze roadway networks using motifs and minimum cycle bases.

The topological characteristics of different layers in urban road networks are shown in Table 2, which include the number of nodes, the number of edges, average degree, average path length, clustering coefficient and network diameter. We find that the average path length and network diameter in the urban road network are larger, and the clustering coefficient is smaller.

### 3.2. Urban Road Analysis Based on Motif

As shown in Table 3, the Z-scores of each motif in the different layers of the road network of Beijing, Shanghai, Guangzhou, Shenzhen, Chengdu and Kunming are summarized. It can be observed that the Z-score of motifs with cyclic structure, such as the Z-scores of 3-2, 4-3 and 4-4 are positive, and their occurrence frequency are significantly higher than that of random networks with sequences of the same degree, while the Z-scores of 3-1, 4-1 and 4-2 are negative. In particular, 4-5 hardly appears in random networks, and 4-6 never appears both in road networks and in random networks. That is to say, the frequency of motifs that contain cycles surpasses that of random networks with equivalent degree sequences. Thus, we conduct topological and robustness analyses of urban road networks using the minimum cycle basis as fundamental components in the following sections.

### 3.3. Urban Road Analysis Based on the Minimum Cycle Basis

The topological characteristics based on the cycle of different layers in the urban road network are shown in Table 4, which include the number of nodes, the number of edges, average degree, the total number of cycles, the average length of the minimum cycle basis, the ratio of nodes in the cycles to total nodes, and the ratio of edges in the cycles to total edges. We can see that the values of $N^{c}/N$ and $M^{c}/M$ are around 80%, indicating that the majority of nodes and edges belong to the minimum cycle basis. Thus, the study of urban roads based on the minimum cycle basis is meaningful.

#### 3.3.1. The Length and Surface Area Distribution of the Minimum Cycle Basis

**Figure 6:**
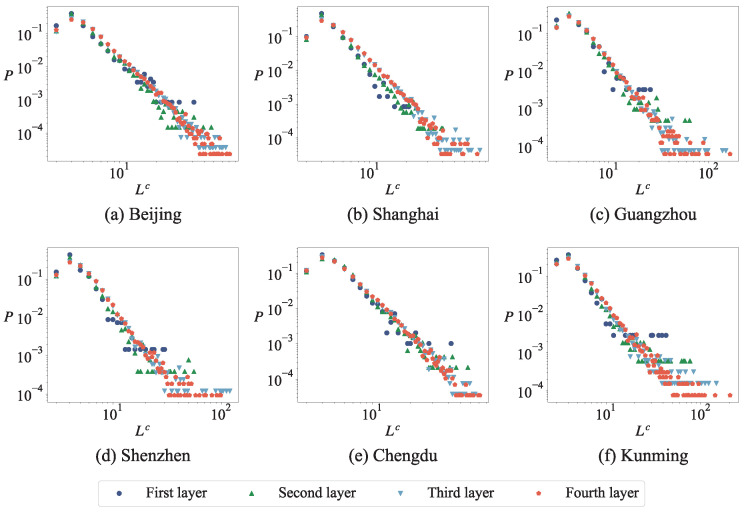
The length distribution of the minimum cycle base in the urban road network for different cities. In all plots, the *x*-axis label $L^{c}$ represents the length of the minimum cycle basis and the *y*-axis label *P* represents the proportion of each part.

**Figure 7:**
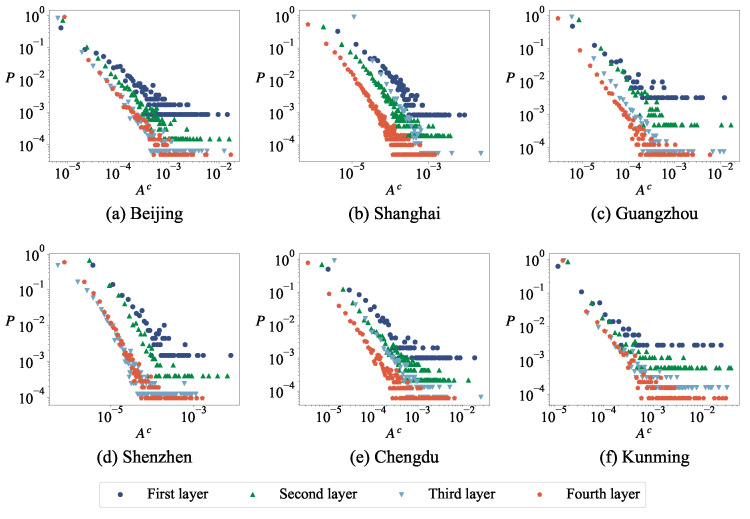
The surface area distribution of the minimum cycle base in the urban road network for different cities. In all plots, the *x*-axis label $A^{c}$ represents the surface areas and the *y*-axis label *P* represents the proportion of each part.

Previous studies have confirmed that the frequency distribution of length and surface areas obey the power-law distribution when the cellular structures or closed polygons are used to study road networks [34,35,36]. Figure 6 and Figure 7 show that the length and surface areas distribution of the minimum cycle basis follows a power-law distribution, with the exception of cycles comprised of three nodes. In other words, in the network, the minimum cycle basis with a smaller length appears more frequently than that with a larger length. Table 5 shows the power index of the distribution of the cycle length of the road network at different layers in six cities, and it can be seen that the power indexes are different in different cities. During the modeling and analysis of urban road networks based on the minimum cycle basis, it is observed that the distribution of lengths and surface areas within the minimum cycle basis does not adhere to uniformity or normality; instead, it follows a power-law distribution. The modeling conducted previously using Voronoi tessellation evidently fails to satisfy this requirement [48].

#### 3.3.2. Vital Nodes Identification Based on the Minimum Cycle Basis

**Figure 8:**
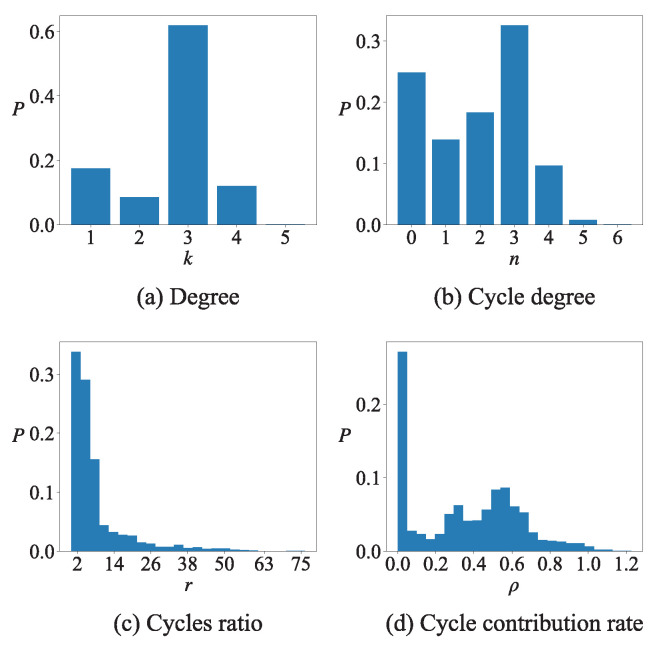
The histogram of four node centrality measurements at the second layer of Kunming road network.

Taking the second layer of the Kunming road network as an example, the value distribution of degree, cycle degree, cycle ratio and cycle contribution rate are also shown in Figure 8. The values of degree and cycle degree are only a few discrete numbers, while the values of cycle ratio and cycle contribution rate have a lot of possibilities. The distribution of cycle ratio exhibits power-law characteristics, while the cycle contribution rate demonstrates normal distribution characteristics. In the context of node ranking, the cycle contribution rate exhibits superior discriminatory power among nodes in comparison to the cycle ratio.

**Figure 9:**
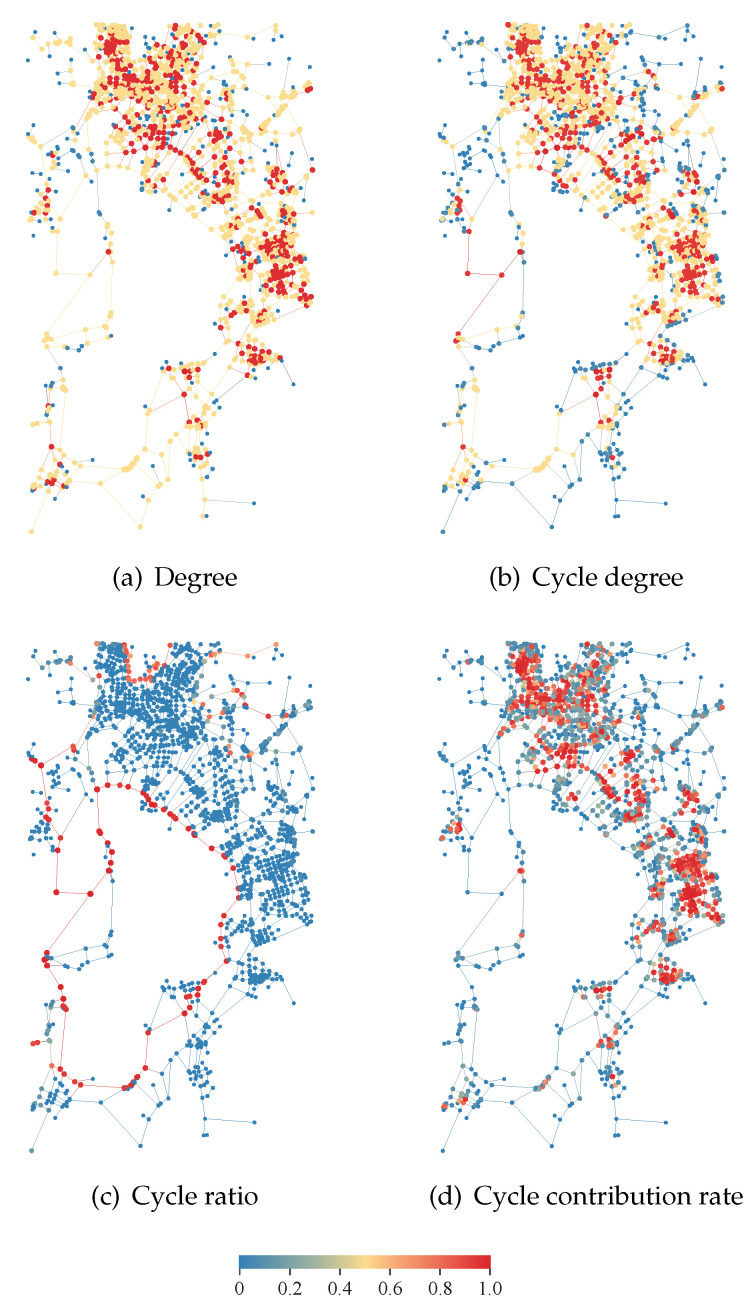
Localized spatial distribution for different normalized node centrality measurements, including degree, cycle degree, cycle ratio and cycle contribution rate, at the second layer of Kunming road network. Node centrality is visually distinguished by color gradients, where colors approaching red indicate higher centrality values and those approaching blue indicate lower values.

The spatial distribution of degree, cycle degree, cycle ratio and cycle contribution rate are shown in Figure 9. Node centrality is visually distinguished by color gradients, where colors approaching red indicate higher centrality values and those approaching blue indicate lower values. Obviously, the nodes are hard to distinguish by degree and cycle degree. Nodes possessing a high cycle ratio are typically situated within larger cycles in Figure 9c. As shown in Figure 9d, nodes with higher cycle contribution rates are typically located in the core area of the network. This result can be used to identify the polycentricity of the network, and this discovery will aid in the selection and identification of urban material hubs and commercial centers.

#### 3.3.3. Robustness Analysis

**Figure 10:**
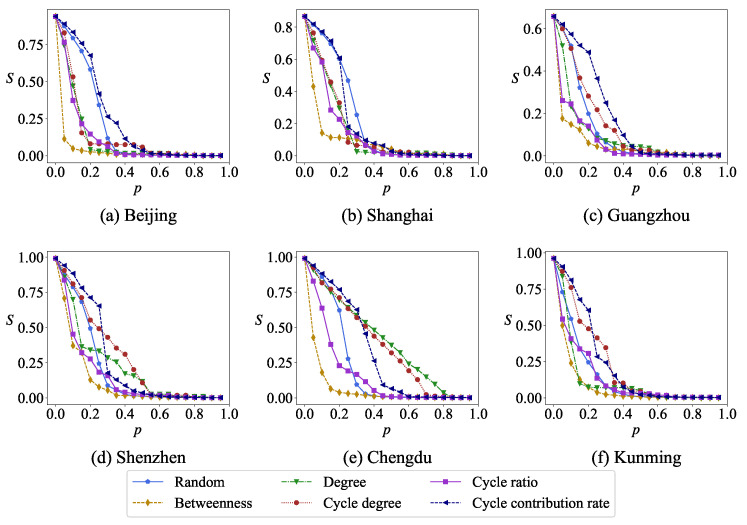
The relative size of the maximum connected subgraph *S* changes with the removal ratio *p* under different removal strategies of nodes.

In order to reduce computing costs, we only selected the second-layer road network of six cities for robustness analysis. In urban road networks, the removal of nodes will affect the changes in network connectivity. Figure 10 shows the relationship between the relative size of the maximum connected subgraph *S* and the proportion of the removed nodes *p* in the same network under six different removing strategies, removing nodes randomly and removing nodes according to degree, betweenness, cycle degree, cycle ratio and cycle contribution rate on network robustness. Obviously, the variation curves of the relative size of the maximum connected subgraph *S* are different under different node removal strategies. The betweenness centrality is relatively sensitive to disruptions in network connectivity, followed by the cycling rate, with the cycle contribution rate being the least sensitive. This may be attributed to the fact that nodes with higher cycle contribution rates are often located in the core areas of the road network, where they enjoy a high degree of connectivity, thus resulting in a weaker impact on network connectivity when disrupted.

**Figure 11:**
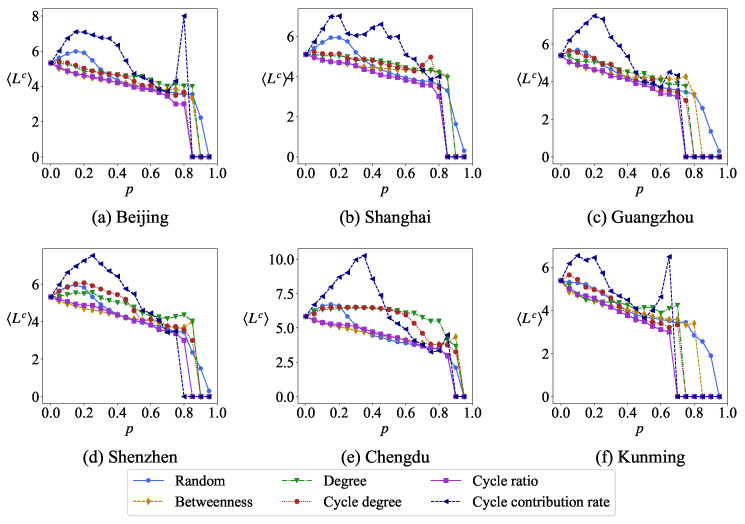
The average length of the minimum cycle basis $\langle L^{c}\rangle$ changes with the removal ratio *p* under different removal strategies.

Removing nodes causes changes in the average length of the minimum cycle basis. Figure 11, respectively, shows the relationship between the average length of the minimum cycle basis $\langle L^{c}\rangle$ and the proportion of removed nodes *p* in the same network under six different removal strategies, removing nodes randomly and removing nodes according to degree, betweenness, cycle degree, cycle ratio and cycle contribution rate on network robustness. Obviously, the variation curves of the average length of the minimum cycle basis $\langle L^{c}\rangle$ are different under different node removal strategies. The change in $\langle L^{c}\rangle$ is more sensitive to the cycle ratio than others. The cycle contribution rate has a relatively minor impact on $\langle L^{c}\rangle$ primarily because small cycles contribute more significantly to the cycle contribution rate, whereas larger cycles contribute more prominently to the cycling rate.

**Figure 12:**
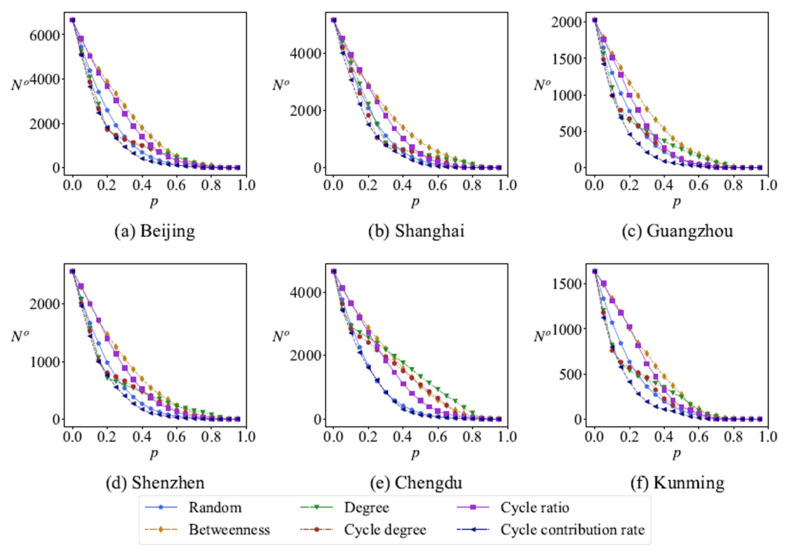
The total number of cycles $N^{o}$ changes with the removing ratio *p* under different removing strategies.

The removal of network nodes will affect the change in the total number of the minimum cycle basis. Figure 12, respectively, shows the relationship between the total number of minimum cycles $N^{o}$ and the proportion of removed nodes *p* in the same network under six different removing strategies, removing nodes randomly and removing nodes according to degree, betweenness, cycle degree, cycle ratio and cycle contribution rate on network robustness. Obviously, the variation curves of the total number of minimum cycles $N^{o}$ are different under different removing strategies of nodes. The change in $N^{o}$ is more sensitive to the cycle contribution rate than others. In a road network of a given area, an increase in the number of cycles indicates superior network connectivity, providing more routing options for navigation and enhancing convenience in daily life. That is to say, the cycle contribution rate characterizes the level of connectivity density within a specific region, functioning as a valuable indicator for assessing the polycentric character of road networks.

In summary, when studying the robustness of the network, with the increase in the number of removed nodes, the betweenness affects the relative size change of the maximum connected subgraph significantly. The cycle ratio affects the average length of the minimum cycle basis significantly. The cycle contribution rate affects the total number of cycles significantly.

### 3.4. Analyzing Cycle-Based Dual Network

**Figure 13:**
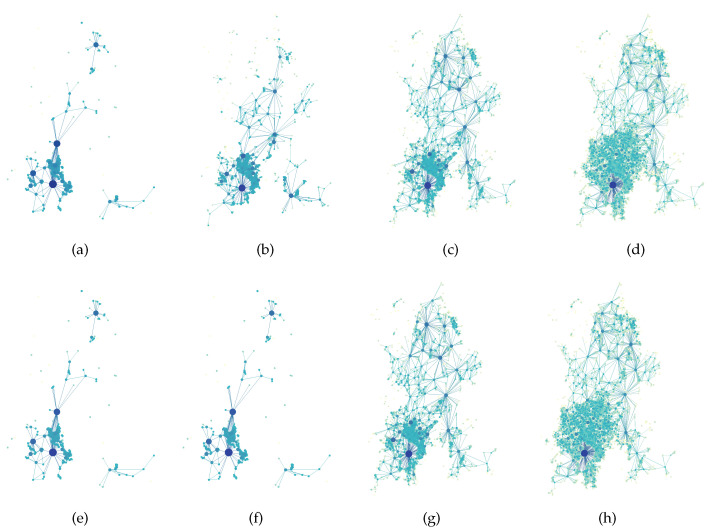
Different layers of cycle-based dual network of Kunming. (**a**–**d**) are the first-fourth layer in sequence and share one node, (**e**–**h**) are the first-fourth layer in sequence and share one edge.

A cycle-based dual network is constructed by treating the smallest cycles as nodes and establishing connections based on shared nodes between cycles. Based on this, we establish two types of cycle-based dual networks: the cycle-based dual network in which the cycles share at least one node and the cycle-based dual network in which the cycles share at least one edge. Taking four layers of the Kunming road network as an example, Figure 13 shows the schematic diagrams of two types of cycle-based dual network. As depicted in the graph, the degree distribution displays power-law properties, characterized by a small proportion of nodes possessing significantly higher degree values. Notably, the node exhibiting the maximum degree value corresponds to the circular roadway encompassing Dianchi Lake.

**Figure 14:**
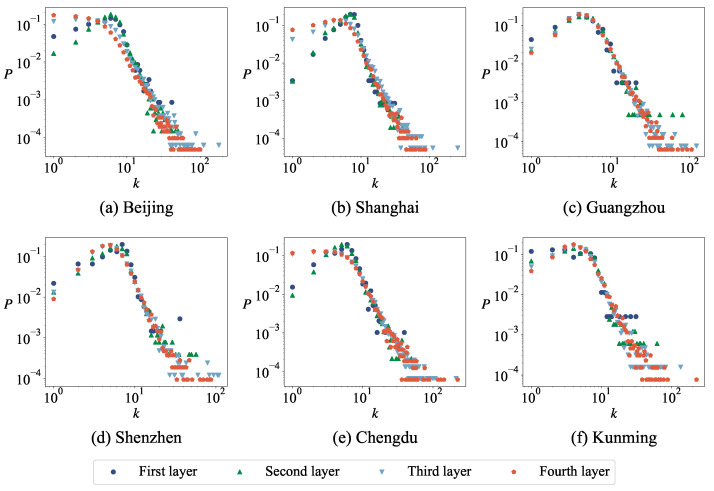
The degree distribution of the cycle-based dual network sharing one node.

**Figure 15:**
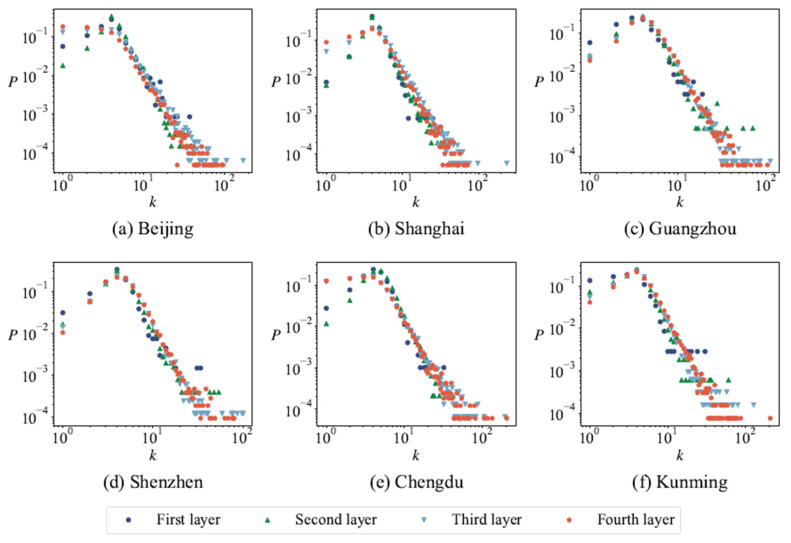
The degree distribution of the cycle-based dual network sharing one edge.

The degree distribution of two kinds of cycle-based dual networks of six cities is shown in Figure 14 and Figure 15, which follow a power-law distribution. In the cycle-based dual network in which the cycles share at least one node, the degree distribution demonstrates power-law behavior when the degree value surpasses 6. Conversely, when the degree value is 6 or below, variations arise across different layers and cities. Similarly, the cycle-based dual network in which the cycles share at least one edge exhibits analogous characteristics, albeit with a modified threshold degree value of 4.

Table 6 and Table 7 illustrate the topological characteristics, including the number of nodes *N*, the number of edges *M*, average degree 〈k〉, average path length 〈d〉, clustering coefficient 〈C〉 and network diameter *D* of two kinds of cycle-based dual networks of six cities, corresponding to Figure 14 and Figure 15, respectively. The results show that two kinds of cycle-based dual networks possess higher average degree and clustering coefficient, and a shorter average path length and network diameter, showing more obvious small-world property compared with the results of urban road networks as shown in Table 2.

In summary, when analyzing urban road networks with the minimum cycle basis serving as the fundamental component, the network displays both small-world and scale-free properties. Notably, the clustering coefficient hovering around 0.5 indicates a specific structure, while the degree distribution of the network conforms to a power-law pattern. As widely recognized, the small-world property signifies a higher degree of interconnectedness and intimacy among nodes, whereas the scale-free property emphasizes the heterogeneity in degree values among nodes, which facilitates the rapid dissemination of information and the distribution of resources. These findings offer a fresh perspective on road network research and implicitly illustrate how the prevalent ring highways in cities, functioning as significant nodes in the cycle-based dual network, can significantly enhance urban living convenience.

## 4. Conclusions

This study focuses on six cities in China(Beijing, Shanghai, Guangzhou, Shenzhen, Chengdu and Kunming) to explore the topological characteristics, the centrality of nodes and the robustness of urban road networks based on motif and the minimum cycle basis. Moreover, in order to display the hierarchical structure of urban roads and reduce the computational complexity, we select four layers of urban road networks for modeling.

Firstly, this study conducted motif analysis on six urban road networks, revealing that motifs containing cycles exhibit positive Z-scores, indicating their prevalence exceeds that of equivalent-degree sequences in random networks; based on this finding, we use the minimum cycle basis as a fundamental element for network analysis. Intriguingly, we observe that the length distribution of the minimum cycle basis in urban road networks follows a power-law distribution; To investigate node importance within urban road networks, we introduce the concept of cycle contribution rate (the importance of a node depends not only on the number of cycles it is associated with but also on the length of these cycles) and compare the results with node degree, cycle degree (the number of cycles associated with a node), and cycle ratio. The findings indicate that nodes with higher cycle degrees typically exhibit higher cycle ratios, while those associated with a greater number of smaller cycles tend to demonstrate higher cycle contributions, underscoring the advantageous role of cycle contribution in identifying central areas within road networks; subsequently, we adopted the relative size of the maximum connected subgraph, the number of cycles in the network, and the average length of cycles as observations to verify the effectiveness of cycle contribution rate in the detection of node importance and compared it with other indicators. The results reveal that with an increasing number of removed nodes, the betweenness centrality significantly impacts the relative size of the maximum connected subgraph, while the cycle ratio notably affects the average length of cycles, and the cycle contribution rate substantially influences the number of cycles; finally, we construct two types of cycle-based dual networks for urban road networks by representing cycles as nodes and establishing edges between two cycles sharing a common node and edge, respectively. The dual network exhibits small-world and scale-free properties, manifested through a power-law degree distribution, high clustering coefficients, and short average path lengths.

The adoption of the minimum cycle basis as a fundamental unit for analyzing urban road networks offers a fresh perspective for associated research endeavors, presenting the following promising applications: (1) In the process of constructing road network generation models, one may incorporate the power-law distribution characteristics of the lengths and areas associated with the minimum cycle basis; (2) by comparing the topological attributes of the minimum cycle basis across diverse cities, existing road networks can be enhanced and optimized; (3) potential applications can be explored, such as utilizing the cycle contribution rate, as introduced in this paper, to delve into the multi-centrality of networks. This metric demonstrates notable performance in both node differentiation and spatial distribution. Future endeavors may further explore related application scenarios, including community detection and link prediction grounded in the minimum cycle basis. Moreover, endeavoring to establish a comprehensive road network description and analysis framework based on the minimum cycle basis will undoubtedly aid in addressing various road-related challenges.

## Figures and Tables

**Table 1:** Node centrality of Figure 2a. *k*: degree; BC: betweeness centrality; *n*: cycle degree; *r*: cycle ratio; ρ: cycle contribute ratio.

Node	Associated Cycles	*k*	BC	*n*	*r*	ρ
$v_{1}$	$O_{1}$	2	0	1	2.5	0.33
$v_{2}$	$O_{1}$	2	0	1	2.5	0.33
$v_{3}$	$O_{2}$	2	18.50	1	4.5	0.14
$v_{4}$	$O_{2}$	2	10.50	1	4.5	0.14
$v_{5}$	$O_{2}$	2	5	1	4.5	0.14
$v_{6}$	Null	1	0	0	0	0
$v_{7}$	$O_{1}$, $O_{3}$	5	51	2	5.33	0.58
$v_{8}$	$O_{2}$, $O_{3}$, $O_{4}$	5	72	3	8.33	0.73
$v_{9}$	$O_{2}$, $O_{4}$, $O_{5}$	3	8.05	3	6.50	0.81
$v_{10}$	$O_{2}$, $O_{5}$, $O_{6}$	3	12.50	3	8.67	0.68
$v_{11}$	$O_{2}$, $O_{6}$	3	9.50	2	7.67	0.35
$v_{12}$	Null	1	0	0	0	0
$v_{13}$	$O_{3}$	3	17	1	2.83	0.25
$v_{14}$	$O_{3}$	2	10	1	2.83	0.25
$v_{15}$	$O_{4}$, $O_{5}$, $O_{6}$	4	29	3	5.17	0.87
$v_{16}$	$O_{6}$	2	10	1	3.17	0.20
$v_{17}$	$O_{6}$	2	2.50	1	3.17	0.20

**Table 2:** The topological characteristics of urban road networks. *N*: the number of nodes; *M*: the number of edges; 〈k〉: average degree; 〈d〉: average path length; 〈C〉: clustering coefficient; *D*: network diameter.

City	Level	*N*	*M*	〈k〉	〈d〉	〈C〉	*D*
Beijing	First layer	3348	4432	2.65	34.97	0.05	106
	Second layer	14,294	20,823	2.91	56.82	0.05	164
	Third layer	65,660	93,949	2.86	138.59	0.05	515
	Fourth layer	102,930	147,576	2.87	165.49	0.06	466
Shanghai	First layer	2110	3265	3.09	23.23	0.04	62
	Second layer	9827	14,960	3.04	45.02	0.04	113
	Third layer	48,723	72,409	2.97	91.58	0.04	229
	Fourth layer	64,201	95,168	2.96	107.73	0.04	266
Guangzhou	First layer	958	1237	2.58	17.48	0.07	40
	Second layer	5096	7053	2.77	38.24	0.06	88
	Third layer	31,796	44,679	2.81	92.39	0.06	245
	Fourth layer	38,416	54,295	2.83	97.47	0.06	271
Shenzhen	First layer	1424	2097	2.95	24.46	0.06	74
	Second layer	5241	7794	2.97	48.44	0.05	129
	Third layer	18,485	26,767	2.90	96.53	0.05	263
	Fourth layer	23,906	34,605	2.90	111.56	0.05	313
Chengdu	First layer	2372	3316	2.80	28.50	0.04	84
	Second layer	10,280	14,907	2.90	51.09	0.05	124
	Third layer	58,947	85,251	2.89	118.49	0.05	320
	Fourth layer	64,966	93,662	2.88	123.51	0.05	331
Kunming	First layer	1310	1629	2.49	36.52	0.07	85
	Second layer	4634	6227	2.69	57.21	0.08	165
	Third layer	17,231	23,411	2.72	94.87	0.07	260
	Fourth layer	33,623	46,411	2.76	115.12	0.08	313

**Table 3:** Z-score for different layers of urban road network.

City	Level	3-1	3-2	4-1	4-2	4-3	4-4	4-5	4-6
Beijing	First layer	−141.70	141.70	−118.07	−63.99	114.26	305.99	*∞*	-
	Second layer	−579.03	579.03	−469.11	−240.63	451.07	1456.05	*∞*	-
	Third layer	−3047.24	3047.24	−2813.78	−756.64	2732.62	5268.11	*∞*	-
	Fourth layer	−4526.36	4526.36	−4086.01	−1226.12	3970.74	7789.24	*∞*	-
Shanghai	First layer	−78.68	78.68	−66.21	−105.91	64.63	260.17	*∞*	-
	Second layer	−253.55	253.55	−228.89	−206.16	224.78	1162.15	*∞*	-
	Third layer	−1782.06	1782.06	−1609.71	−611.13	1571.48	5186.93	*∞*	-
	Fourth layer	−2083.12	2083.12	−1937.58	−924.20	1897.10	5609.74	*∞*	-
Guangzhou	First layer	−54.12	54.12	−46.51	−36.29	46.02	62.40	*∞*	-
	Second layer	−268.79	268.79	−245.67	−128.32	237.82	447.30	*∞*	-
	Third layer	−1666.81	1666.81	−1597.69	−578.39	1557.39	2611.52	*∞*	-
	Fourth layer	−1779.24	1779.24	−1718.14	−659.79	1671.67	2828.27	*∞*	-
Shenzhen	First layer	−67.63	67.63	−56.45	−77.69	53.23	169.11	*∞*	-
	Second layer	−253.92	253.92	−224.53	−164.04	217.47	504.77	*∞*	-
	Third layer	−895.10	895.10	−821.19	−478.17	792.83	1735.01	*∞*	-
	Fourth layer	−1042.45	1042.45	−973.23	−692.74	941.60	2052.26	*∞*	-
Chengdu	First layer	−84.39	84.39	−79.59	−70.49	78.08	211.80	*∞*	-
	Second layer	−364.80	364.80	−334.94	−341.94	326.87	917.95	*∞*	-
	Third layer	−2500.57	2500.57	−2238.12	−760.30	2183.14	5054.40	*∞*	-
	Fourth layer	−2807.33	2807.33	−2521.32	−852.74	2457.29	5838.93	*∞*	-
Kunming	First layer	−78.17	78.17	−68.40	−40.97	63.27	91.25	*∞*	-
	Second layer	−275.56	275.56	−241.95	−118.20	227.13	350.02	*∞*	-
	Third layer	−1199.16	1199.16	−1064.96	−329.96	1009.31	1445.67	*∞*	-
	Fourth layer	−1957.88	1957.88	−1854.06	−672.93	1768.70	3073.76	*∞*	-

**Table 4:** The topological characteristics associated with cycles of the urban road network. *N*: the number of nodes; *M*: the number of edges; 〈k〉: average degree; $N^{o}$: the total number of cycles; $\langle L^{c}\rangle$: the average length of the minimum cycle basis; $N^{c}$: the number of nodes within cycles; $M^{c}$: the number of edges within cycles; $N^{c}/N$: the ratio of nodes in the cycles to total nodes; $M^{c}/M$: the ratio of edges in the cycles to total edges.

City	Level	*N*	*M*	〈k〉	$N^{o}$	$\langle L^{c}\rangle$	$N^{c}/N$	$M^{c}/M$
Beijing	First layer	3348	4432	2.65	1167	5.23	0.69	0.77
	Second layer	14,294	20,823	2.91	6656	5.32	0.84	0.89
	Third layer	65,660	93,949	2.86	27,170	5.87	0.81	0.85
	Fourth layer	102,930	147,576	2.87	42,887	5.93	0.84	0.87
Shanghai	First layer	2110	3265	3.09	1175	4.80	0.84	0.90
	Second layer	9827	14,960	3.04	5173	5.10	0.86	0.91
	Third layer	48,723	72,409	2.97	23,022	5.77	0.87	0.90
	Fourth layer	64,201	95,168	2.96	29,936	5.85	0.88	0.91
Guangzhou	First layer	958	1237	2.58	305	5.12	0.66	0.75
	Second layer	5096	7053	2.77	2029	5.39	0.79	0.85
	Third layer	31,796	44,679	2.81	13,019	5.83	0.87	0.90
	Fourth layer	38,416	54,295	2.83	16,029	5.87	0.88	0.91
Shenzhen	First layer	1424	2097	2.95	684	4.99	0.83	0.89
	Second layer	5241	7794	2.97	2569	5.32	0.89	0.93
	Third layer	18,485	26,767	2.90	8312	5.79	0.91	0.94
	Fourth layer	23,906	34,605	2.90	10,753	5.84	0.92	0.95
Chengdu	First layer	2372	3316	2.80	979	5.40	0.80	0.86
	Second layer	10,280	14,907	2.90	4658	5.83	0.92	0.94
	Third layer	58,947	85,251	2.89	26,618	5.91	0.88	0.91
	Fourth layer	64,966	93,662	2.88	28,755	5.97	0.87	0.91
Kunming	First layer	1310	1629	2.49	351	5.14	0.62	0.69
	Second layer	4634	6227	2.69	1642	5.38	0.75	0.81
	Third layer	17,231	23,411	2.72	6340	5.84	0.81	0.86
	Fourth layer	33,623	46,411	2.76	13,051	5.98	0.85	0.89

**Table 5:** The power-law exponent of the length distribution.

City	First Layer	Second Layer	Third Layer	Fourth Layer
Beijing	3.43	4.08	3.62	3.64
Shanghai	4.15	3.88	3.76	3.83
Guangzhou	3.69	3.65	3.55	3.52
Shenzhen	3.78	3.96	3.85	3.78
Chengdu	4.14	3.86	3.59	3.56
Kunming	3.53	3.35	3.10	3.12

**Table 6:** The topological characteristics of cycle-based dual network sharing one node. $N^{v}$: the number of nodes; $M^{v}$: the number of edges; $\langle k^{v}\rangle$: average degree; $C^{v}$: the number of connected subgraphs; $N_{max}^{v}$: the number of nodes in the maximal connected subgraph; $\langle d^{v}\rangle$: average path length of the maximal connected subgraph; $\langle C^{v}\rangle$: clustering coefficient of the maximal connected subgraph; $D^{v}$: network diameter of the maximal connected subgraph.

City	Level	$N^{v}$	$M^{v}$	$\langle k^{v}\rangle$	$C^{v}$	$N_{\max}^{v}$	$\langle d^{v}\rangle$	$\langle C^{v}\rangle$	$D^{v}$
Beijing	First layer	1167	3428	5.87	43	994	9.88	0.54	28
	Second layer	6656	21,285	6.40	91	6435	16.27	0.52	46
	Third layer	27,170	87,385	6.43	175	24,804	27.21	0.55	79
	Fourth layer	42,887	133,913	6.24	269	39,864	33.71	0.55	89
Shanghai	First layer	1175	4082	6.95	3	1102	9.97	0.51	23
	Second layer	5173	17,437	6.74	12	4605	15.56	0.51	39
	Third layer	23,022	79,227	6.88	42	19,634	22.40	0.54	59
	Fourth layer	29,936	99,225	6.63	50	26,080	27.35	0.54	82
Guangzhou	First layer	305	764	5.01	13	159	6.19	0.57	17
	Second layer	2029	5758	5.68	42	1216	7.01	0.57	18
	Third layer	13,019	36,649	5.63	193	8149	12.69	0.56	43
	Fourth layer	16,029	45,634	5.69	188	10,531	16.91	0.56	52
Shenzhen	First layer	684	2132	6.23	9	594	6.82	0.53	16
	Second layer	2569	7979	6.21	14	2122	10.69	0.53	26
	Third layer	8312	24,304	5.85	36	8224	16.00	0.54	46
	Fourth layer	10,753	31,256	5.81	39	10,617	19.17	0.55	61
Chengdu	First layer	979	2869	5.86	11	915	9.06	0.52	29
	Second layer	4658	13,834	5.94	26	4594	13.08	0.53	32
	Third layer	26,618	87,331	6.56	144	26,288	27.48	0.55	77
	Fourth layer	28,755	93,768	6.52	153	28,396	28.76	0.55	73
Kunming	First layer	351	830	4.73	30	256	5.66	0.51	13
	Second layer	1642	4432	5.40	80	1343	7.69	0.53	22
	Third layer	6340	17,482	5.51	173	5951	10.91	0.55	29
	Fourth layer	13,051	36,717	5.63	266	12,407	13.23	0.57	37

**Table 7:** The topological characteristics of cycle-based dual network sharing one edge. $N^{e}$: the number of nodes; $M^{e}$: the number of edges; $\langle k^{e}\rangle$: average degree; $C^{e}$: the number of connected subgraphs; $N_{max}^{e}$: the number of nodes in the maximal connected subgraph; $\langle d^{e}\rangle$: average path length of the maximal connected subgraph; $\langle C^{e}\rangle$: clustering coefficient of the maximal connected subgraph; $D^{e}$: network diameter of the maximal connected subgraph.

City	Level	$N^{e}$	$M^{e}$	$\langle k^{e}\rangle$	$C^{e}$	$N_{\max}^{e}$	$\langle d^{e}\rangle$	$\langle C^{e}\rangle$	$D^{e}$
Beijing	First layer	1167	2560	4.39	45	989	10.80	0.39	31
	Second layer	6656	16,401	4.93	94	6432	17.74	0.40	52
	Third layer	27,170	77,248	5.69	185	24,780	28.37	0.49	83
	Fourth layer	42,887	122,138	5.70	287	39,832	35.25	0.51	93
Shanghai	First layer	1175	2670	4.54	4	1102	12.10	0.28	28
	Second layer	5173	12,509	4.84	14	4597	17.57	0.35	47
	Third layer	23,022	66,741	5.80	44	19,624	24.06	0.46	67
	Fourth layer	29,936	86,875	5.80	53	26,078	28.76	0.48	83
Guangzhou	First layer	305	606	3.97	15	132	5.03	0.41	13
	Second layer	2029	4772	5.68	44	1214	7.40	0.46	19
	Third layer	13,019	33,701	5.18	198	8141	13.10	0.52	45
	Fourth layer	16,029	42,196	5.27	197	10,515	17.72	0.52	56
Shenzhen	First layer	684	1519	4.44	9	594	7.93	0.34	21
	Second layer	2569	6302	4.91	15	2122	11.73	0.41	28
	Third layer	8312	22,207	5.34	41	8217	16.41	0.49	46
	Fourth layer	10,753	28,996	5.39	41	10,616	19.47	0.51	61
Chengdu	First layer	979	2348	4.80	12	912	10.13	0.43	33
	Second layer	4658	12709	5.46	30	4589	13.54	0.49	33
	Third layer	26,618	76,593	5.76	156	26,268	29.42	0.49	81
	Fourth layer	28,755	83,576	5.81	161	28,385	30.39	0.50	76
Kunming	First layer	351	638	3.64	30	256	6.23	0.38	15
	Second layer	1642	3612	4.40	83	1309	8.05	0.43	22
	Third layer	6340	15,608	4.92	180	5943	11.34	0.50	31
	Fourth layer	13,051	34,001	5.21	272	12,400	13.47	0.54	38

## Data Availability

The data that support the findings of this study are available upon reasonable request from the corresponding author. The codes and partial data have been posted at https://github.com/kust-yangbo/Urban-Road-Networks/tree/master.
